# Protective behavior and SARS-CoV-2 infection risk in the population – Results from the Gutenberg COVID-19 study

**DOI:** 10.1186/s12889-022-14310-6

**Published:** 2022-10-31

**Authors:** Rieke Baumkötter, Simge Yilmaz, Daniela Zahn, Katharina Fenzl, Jürgen H. Prochaska, Heidi Rossmann, Irene Schmidtmann, Alexander K. Schuster, Manfred E. Beutel, Karl J. Lackner, Thomas Münzel, Philipp S. Wild

**Affiliations:** 1 Preventive Cardiology and Preventive Medicine, Center for Cardiology, University Medical Center of the Johannes Gutenberg University Mainz, Langenbeckstr. 1, 55131 Mainz, Germany; 2 German Center for Cardiovascular Research (DZHK), partner site Rhine Main, Mainz, Germany; 3 Center for Thrombosis and Hemostasis (CTH), University Medical Center of the Johannes Gutenberg University Mainz, Mainz, Germany; 4 Institute of Clinical Chemistry and Laboratory Medicine, University Medical Center of the Johannes Gutenberg University Mainz, Mainz, Germany; 5 Institute of Medical Biostatistics, Epidemiology and Informatics, University Medical Center of the Johannes Gutenberg University Mainz, Mainz, Germany; 6 Department of Ophthalmology, University Medical Center of the Johannes Gutenberg University Mainz, Mainz, Germany; 7 Department of Psychosomatic Medicine, University Medical Center of the Johannes Gutenberg University Mainz, Mainz, Germany; 8 Cardiology I, Center for Cardiology, University Medical Center of the Johannes Gutenberg University Mainz, Mainz, Germany; 9 Institute of Molecular Biology (IMB), Mainz, Germany

**Keywords:** SARS-CoV-2, COVID-19, pandemics, preventive measures

## Abstract

**Background:**

During the SARS-CoV-2 pandemic, preventive measures like physical distancing, wearing face masks, and hand hygiene have been widely applied to mitigate viral transmission. Beyond increasing vaccination coverage, preventive measures remain urgently needed. The aim of the present project was to assess the effect of protective behavior on SARS-CoV-2 infection risk in the population.

**Methods:**

Data of the Gutenberg COVID-19 Study (GCS), a prospective cohort study with a representative population-based sample, were analyzed. SARS-CoV-2 infections were identified by sequential sampling of biomaterial, which was analyzed by RT-qPCR and two antibody immunoassays. Self-reported COVID-19 test results were additionally considered. Information on protective behavior including physical distancing, wearing face masks, and hand hygiene was collected via serial questionnaire-based assessments. To estimate adjusted prevalence ratios and hazard ratios, robust Poisson regression and Cox regression were applied.

**Results:**

In total, 10,250 participants were enrolled (median age 56.9 [43.3/68.6] years, 50.8% females). Adherence to preventive measures was moderate for physical distancing (48.3%), while the use of face masks (91.5%) and the frequency of handwashing (75.0%) were high. Physical distancing appeared to be a protective factor with respect to SARS-CoV-2 infection risk independent of sociodemographic characteristics and individual pandemic-related behavior (prevalence ratio [PR] = 0.77, 95% confidence interval [CI] 0.62–0.96). A protective association between wearing face masks and SARS-CoV-2 transmission was identified (PR = 0.73, 95% CI 0.55–0.96). However, the protective effect declined after controlling for potential confounding factors (PR = 0.96, 95% CI 0.68–1.36). For handwashing, this investigation did not find a beneficial impact. The adherence to protective behavior was not affected by previous SARS-CoV-2 infection or immunization against COVID-19.

**Conclusion:**

The present study suggests primarily a preventive impact of physical distancing of 1.5 m, but also of wearing face masks on SARS-CoV-2 infections, supporting their widespread implementation. The proper fit and use of face masks are crucial for effectively mitigating the spread of SARS-CoV-2 in the population.

**Supplementary information:**

The online version contains supplementary material available at 10.1186/s12889-022-14310-6.

## Background

During the ongoing severe acute respiratory syndrome coronavirus 2 (SARS-CoV-2) pandemic, preventive measures such as physical distancing, wearing face masks, and hand hygiene have been widely applied to mitigate the burden of coronavirus disease 19 (COVID-19). Countries have taken specific measures depending on the course of the pandemic and the prevailing variant, including closing public life during periods of high incidence of SARS-CoV-2. A study using a database of global pandemic measures showed that countries’ responses to the pandemic were similar in the early post-outbreak period and varied thereafter in 2020 [1]. In Germany, the government recommended keeping physical distance from others, limiting personal contact, and washing hands thoroughly from the beginning of the pandemic. As of the end of April 2020, it was mandatory to wear a face mask indoors and on public transportation [2, 3]. Although COVID-19 vaccination programs have been successfully implemented in most countries, protective behavior remains urgently necessary since vaccination coverage rates are not complete and transmission of SARS-CoV-2 infection remains possible despite vaccination [4–6].

International evidence predominantly suggests that preventive measures such as keeping distance from others, closing public facilities, and use of face masks are effective in reducing viral transmission [7–11]. However, evidence on protective measures for prevention of SARS-CoV-2 is mostly derived from simulation or ecological studies [12–15]. Besides, systematic reviews considered research on the impact of wearing face masks as insufficient, particularly in community settings [10, 16]. Hence, there is still a paucity of evidence on this topic and results are inconclusive. More generally, studies replicating the findings of personal protective behavior and its impact on mitigating SARS-CoV-2 transmission with large population cohorts including individual-level data are limited in literature.

Hence, the present study aimed to investigate the effect of protective behavior, including physical distancing, wearing face masks, and hand hygiene on SARS-CoV-2 infection risk in the population by making use of the Gutenberg COVID-19 Study: a large prospective cohort of individuals sampled from the population during the second and third wave of the SARS-CoV-2 pandemic in Germany in a representative manner.

## Methods

### Study design and population sample

The data used to address the objective are part of the Gutenberg COVID-19 Study (GCS), a population-based prospective cohort study. This study includes a representative sample of individuals aged 25 to 88 years which was randomly drawn by the regional registration offices. The sample was stratified in a 1:1 manner by sex and place of residence (City of Mainz/District of Mainz-Bingen), as well as for age decades. Apart from residence in the City of Mainz or the District of Mainz-Bingen, ability to visit the study center, and sufficient understanding of the German language, no inclusion or exclusion criteria were defined. The baseline examination was conducted from October 2020 to April 2021, and the follow-up assessment approximately four months later from March 2021 to June 2021. At both baseline and follow-up, a computer-assisted personal interview was performed and biomaterial was sampled at the study center. Additionally, participants were sent questionnaires in advance to return to the study site. In addition to biomaterial sampling, the highly granular data collection included SARS-CoV-2 infection, symptoms, protective behaviors, comorbidities, health-related risk factors, medical care, psychosocial burden, and lifestyle and environmental factors. Regarding demographic characteristics, detailed data on age, sex, marital status, children, education, occupation, migration background, and working and living conditions were collected as part of the study. The GCS was designed, conducted, and analyzed in accordance with the Declaration of Helsinki and Good Epidemiological Practice.

### Identification of SARS-CoV-2 infections in the sample

SARS-CoV-2 infections were identified using a multimodal approach in which biomaterial was collected at baseline and follow-up. To identify acute infection, throat swabs were analyzed by quantitative reverse transcription polymerase chain reaction (RT-qPCR) using the Light Mix SarbecoV E-gene (plus EAV control) and RdRP-gene (TIB Molbiol, Germany). The methods used for RT-qPCR have been described elsewhere (laboratory 2) [17]. EDTA plasma samples were analyzed for circulating antibodies to the SARS-CoV-2 nucleocapsid with a qualitative microparticle chemiluminescent immunoassay (Architect SARS-CoV-2 IgG, Abbott, Germany) with a threshold of 1.4 relative light units and second, a qualitative microparticle electro-chemiluminescence immunoassay (Elecsys Anti-SARS-CoV-2 Pan-Ig, Roche, Germany) with a cutoff index of 0.8. Any self-reported PCR results were additionally included which were collected retrospectively via a computer-assisted personal interview and continuously via a weekly smartphone-app survey. Individuals were considered infected if any of these measurements were positive. Detailed information on sample preprocessing and storage is provided in the **Supplementary Appendix**.

### Assessment of protective behavior

Information on protective behavior was collected twice, at baseline and follow-up, using questionnaires. In terms of physical distance, study participants were asked how often they maintained a minimum distance of 1.5 m from contacts outside their household. Regarding the use of face masks, they were asked how often they wore masks (surgical masks, N95/FFP2 respirators, or self-made masks without differentiation) e.g., while shopping, working, or on public transportation. For both questions, answer options ranged from “never” to “always/almost always” on a 5-point Likert scale. Compliance with physical distancing and wearing masks was defined when participants indicated they adhered “almost always/always”. Frequency of hand washing was classified according to published literature with 0–5 times per day as rarely, 6–10 times as moderate, and > 10 times as frequent [11, 18]. Adherence to hand hygiene was defined when handwashing was reported as at least moderate.

### Statistical analysis

Continuous variables were summarized by median and interquartile range. Absolute and relative frequencies were calculated for categorical variables.

Prior to analysis, a directed acyclic graph (DAG) was generated using DAGitty software [19]. The minimum adjustment set based on the DAG comprised age, sex, socioeconomic status (SES), time of enrollment into study, occupational status, and pandemic-related behaviors (COVID-19 vaccination status, COVID-19 contact in the past two weeks, participation in gatherings, travel in the past two weeks, hand disinfection, and avoidance of shaking hands, attending gatherings, and hugging of people in direct surrounding area).

Robust Poisson regression models with adjustment for potential confounders were applied to evaluate the effect of protective behavior on SARS-CoV-2 infection risk. A time-to-event analysis was performed to investigate the risk of incident SARS-CoV-2 infection as a function of protective behavior. Kaplan-Meier estimators were used to generate cumulative incidence plots, and log-rank tests to test for differences between curves. Univariate and multivariable Cox regression models were used to estimate the effect of protective behavior on incident SARS-CoV-2 infections from January 2020 to June 2021. Before October 2020 (i.e., before the study start), only retrospectively collected self-reported infections were considered. Participants without infection date, who were primarily individuals identified as infected with SARS-CoV-2 via positive antibody measurements only, were excluded in time-to-event analysis. Finally, an interaction analysis was performed within an adjusted robust Poisson regression to examine whether the effect of protective behavior on SARS-CoV-2 infections was moderated by age. Protective behavior at baseline was considered as exposure to infection and was assumed not to change. Complete case analyses were performed for all regression models.

In this exploratory approach, p-values were used as continuous measures of statistical evidence of an effect. All analyses were carried out using the software package R version 4.1.0 [20].

## Results

### Characteristics of the population sample

A total of 10,250 participants with a median age of 56.9 (43.3/68.6) years and a balanced sex ratio (50.8% female participants) were enrolled in the GCS. The majority were Caucasian (99.3%). Sociodemographic characteristics and pandemic-related behavior of the sample stratified by sex are shown in Table 1. Women were slightly younger than men and more likely employed. Conversely, women had lower SES compared with males. Adherence to physical distancing was moderate, while use of face masks and frequency of handwashing were high, which was true for both sexes. Women were more likely than men to engage in protective behaviors and showed less travel activities and higher COVID-19 vaccination rates.

**Table 1:** Characteristics of the population sample

	Men n = 5,044	Women n = 5,204
**Sociodemographics**		
Age [years] (IQR)	58.9 (44.9/70.2)	55.4 (41.6/66.8)
SES (IQR)	16.0 (12.0/19.0)	14.0 (11.0/18.0)
Employed [%] (n)	59.8 (2,596)	60.8 (2,792)
**Protective behavior**		
*Adherence to physical distancing*		
Never [%] (n)	0.2 (9)	0.1 (5)
Rarely [%] (n)	0.9 (41)	0.5 (26)
Sometimes [%] (n)	3.4 (160)	2.5 (122)
Often [%] (n)	49.9 (2,342)	46.1 (2,288)
Almost always/always [%] (n)	45.7 (2,145)	50.8 (2,526)
*Adherence to wearing face masks*		
Never [%] (n)	0 (0)	0.1 (4)
Rarely [%] (n)	0.2 (9)	0.1 (6)
Sometimes [%] (n)	0.5 (22)	0.1 (5)
Often [%] (n)	10.8 (507)	5.3 (264)
Almost always/always [%] (n)	88.5 (4,157)	94.4 (4,704)
Washing hands [n/day] (IQR)	7.5 (5.0/10.0)	10.0 (6.0/12.0)
Disinfecting hands [n/day] (IQR)	2.0 (1.0/4.0)	3.0 (1.0/5.0)
COVID-19 vaccination [%] (n)	3.4 (160)	5.5 (274)
Participation in gatherings [%] (n)	11.4 (537)	10.1 (502)
Participation in large gatherings* [%] (n)	3.3 (155)	3.4 (169)
Avoidance of shaking hands^#^ [%] (n)	75.9 (3,533)	77.0 (3,806)
*Travel*		
Domestic [%] (n)	39.6 (1,856)	28.0 (1,386)
Foreign [%] (n)	1.2 (58)	0.6 (29)

Presented are relative and absolute frequencies or medians with interquartile ranges

* Large gatherings refer to gatherings where at least ten people were present

^#^ of people in direct surrounding area

SES, socioeconomic status; IQR, interquartile range

### Prevalence and incidence of SARS-CoV-2 infection in relation to adherence to protective measures

**Fig. 1:**
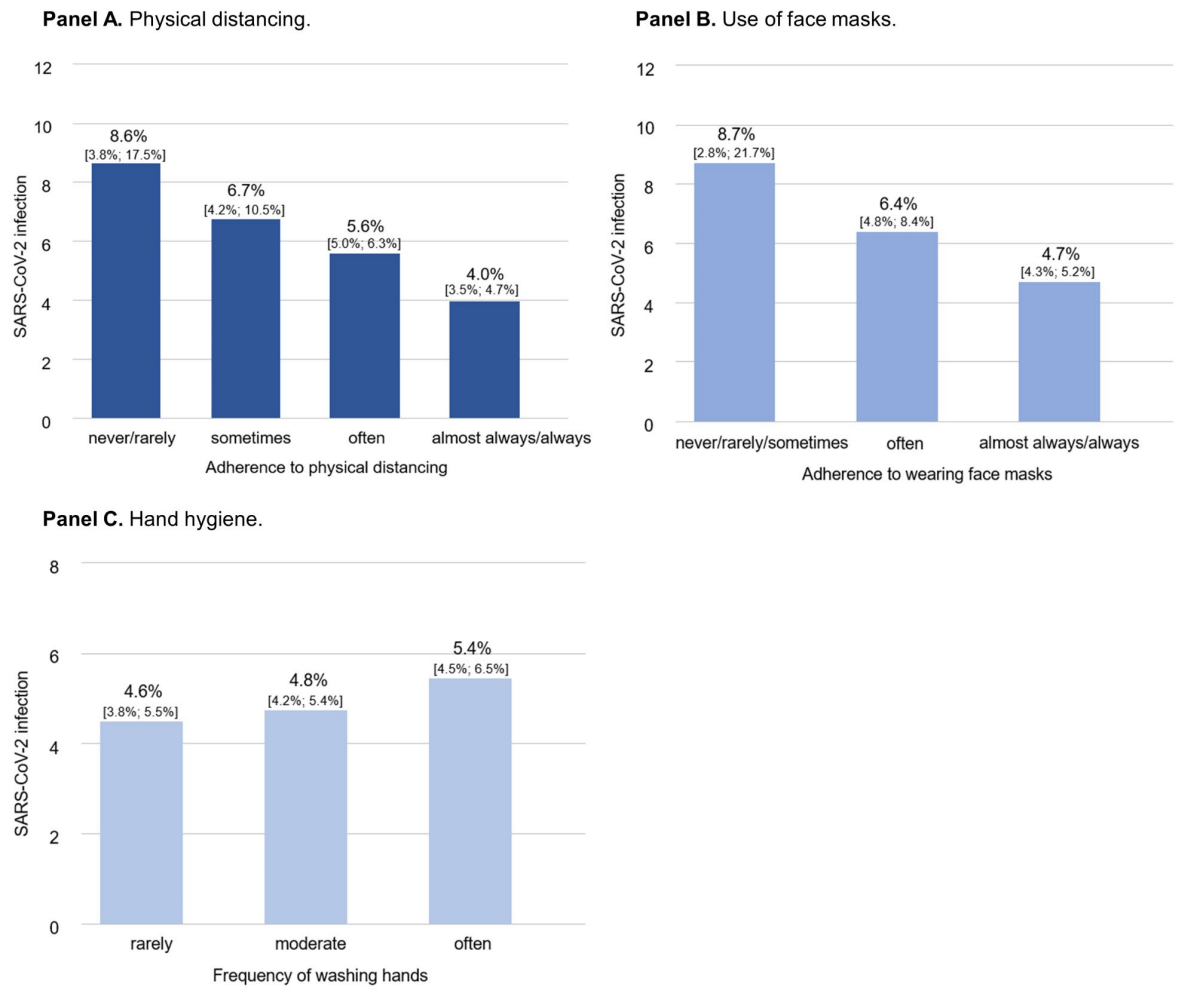
SARS-CoV-2 infection risk according to protective behavior in the population sample. The figure illustrates the prevalence of SARS-CoV-2 infection as a function of physical distancing (n = 9,664), use of face masks (n = 9,678), and hand hygiene (n = 9,326), respectively.

Among individuals who never or rarely maintained the minimum distance, the prevalence of SARS-CoV-2 infection was approximately twice that of persons who almost always or always maintained a physical distance (Fig. 1, **Panel A**). A similar trend was observed for face mask use: Substantially fewer infections were found in individuals who almost always or always wore a face mask than in individuals who did not (Fig. 1, **Panel B**). Regarding hand hygiene, no relevant protective association with SARS-CoV-2 infection risk was observed (Fig. 1, **Panel C)**.

**Fig. 2:**
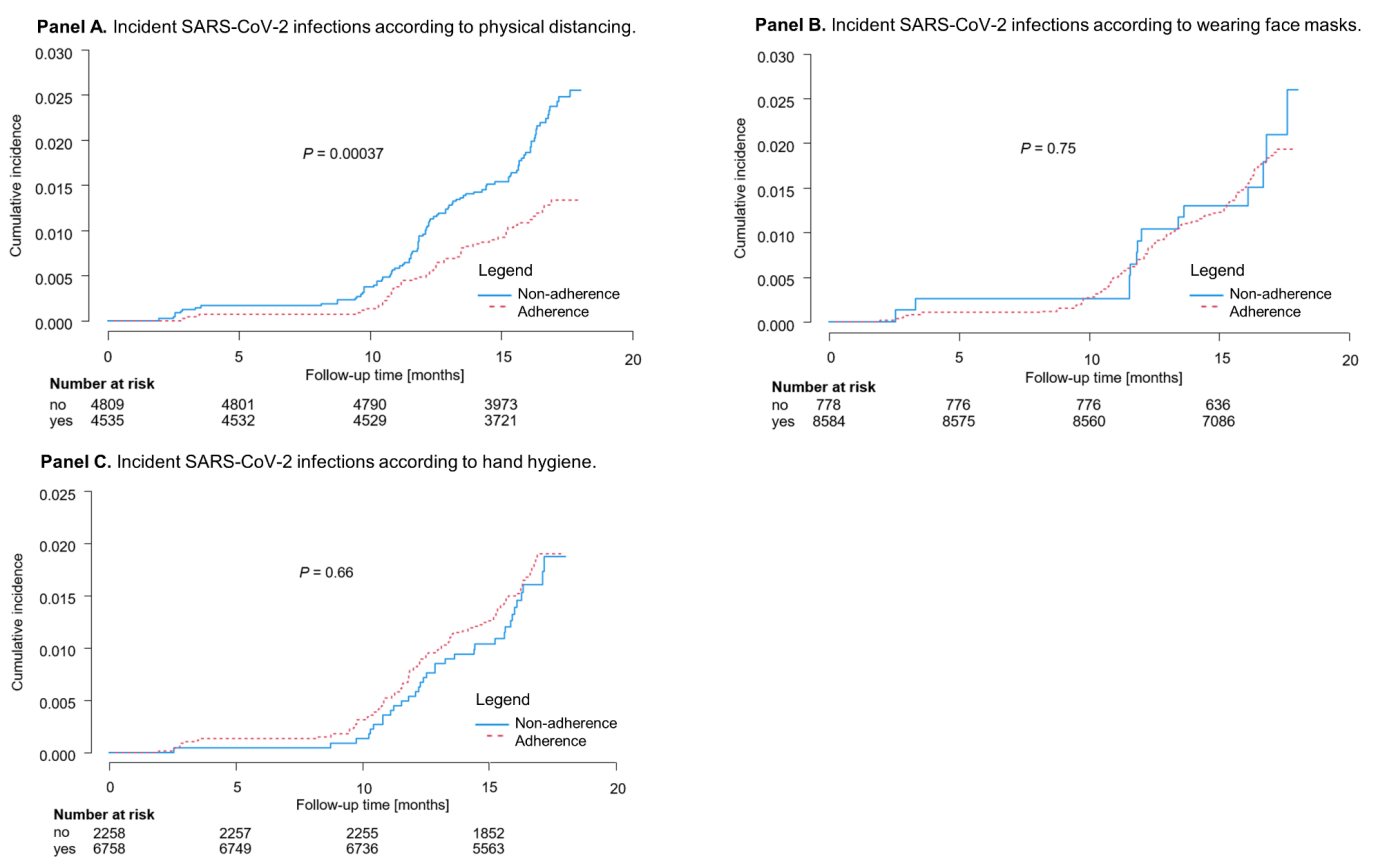
Incident SARS-CoV-2 infections corresponding to protective behavior from January 2020 to June 2021. This figure presents the cumulative incidence of SARS-CoV-2 infections in the population sample corresponding to the adherence to physical distancing (n = 9,344), use of face masks (n = 9,362), and hand hygiene (n = 9,016), respectively. Prior to October 2020 (i.e., the start of the GCS), only self-reported positive PCR tests were considered. Individuals with antibody positivity only were excluded

The estimated cumulative incidence of SARS-CoV-2 infections in the study region between January 2020 to June 2021 is shown in Fig. 2, assuming no change in protective behavior, which was so confirmed in the study cohort. While individuals who adhered to physical distancing had a cumulative incidence of 1.3% after 18 months, a cumulative incidence of 2.6% was observed among non-adherent individuals (log-rank *P* = 0.00037, Fig. 2, **Panel A**). Regarding the use of face masks (log-rank *P* = 0.75) and hand hygiene (log-rank *P* = 0.66), no relevant differences in the incidence of SARS-CoV-2 infection were observed in adherent and non-adherent individuals (Fig. 2, **Panel B and C**).

### Estimating the impact of protective behaviors on SARS-CoV-2 infection risk

Physical distancing (prevalence ratio [PR] = 0.71, 95% confidence interval [CI] 0.59–0.85) and use of face masks (PR = 0.73, 95% CI 0.55–0.96) were found to have a protective effect on reducing the risk of SARS-CoV-2 infections in univariate robust Poisson regression. While physical distancing remained an independent protective factor associated with a 23% (PR = 0.77, 95% CI 0.62–0.96) reduction in SARS-CoV-2 infection risk even after adjusting for time of entry into the study, sociodemographic characteristics, and individual pandemic-related behaviors, the estimated effect of wearing face masks was smaller. No beneficial effect on SARS-CoV-2 infection risk was observed regarding the frequency of hand washing (Table 2).

Cox regression models estimated that physical distancing reduced infection risk by 44% (hazard ratio [HR] = 0.66, 95% CI 0.40–0.77). However, after adjustment for time of enrolment into study, demographic characteristics, and pandemic-related behaviors, the effect size was slightly attenuated. Regarding the use of face masks and hand hygiene, there was no evidence of a beneficial impact on the incidence of SARS-CoV-2 infections in univariate or adjusted models (**Supplemental Table 1**).

To evaluate the impact of protective behavior during the time course of the COVID-19 pandemic, analyses were stratified by the second and third SARS-CoV-2 infection wave in Germany. Results did not differ significantly during the pandemic (**Supplemental Fig. 1**). The effect of protective behaviors on SARS-CoV-2 infection risk did also not meaningfully differ between time periods when either the wild type or alpha variant was dominant in Germany (**Supplemental Table 2**). Finally, an interaction analysis to explore whether older individuals, a particularly vulnerable group at risk for a severe course of COVID-19, benefited disproportionately from protective behavior revealed no moderation by age (*P* for interaction = 0.81; **Supplemental Fig. 2**).

**Table 2:** Effect of protective behavior on SARS-CoV-2 infection risk in the population sample

	Univariate analysis*		Adjusted for time of enrollment, sociodemographics, pandemic-related behavior^†^	
	Prevalence ratio [95% CI]	*P*	Prevalence ratio [95% CI]	*P*
Physical distancing	0.71 [0.59; 0.85]	0.00015	0.77 [0.62; 0.96]	0.022
Use of face masks	0.73 [0.55; 0.96]	0.025	0.96 [0.68; 1.36]	0.83
Hand hygiene	1.08 [0.88; 1.34]	0.46	1.12 [0.88; 1.44]	0.36

Robust Poisson regression models. Dependent variable: SARS-CoV-2 infections during baseline and follow-up; independent variables: physical distancing, wearing face masks, and hand hygiene at baseline

* Physical distancing (n = 9,664), use of face masks (n = 9,678), hand hygiene

(n = 9,326)

^†^ Adjustment for time of enrolment into study, age, sex, socioeconomic status, occupational status, COVID-19 vaccination status, COVID-19 contact in the past two weeks, participation in gatherings in the past two weeks, travel (any), disinfecting hands (> 2 per day), and avoidance of shaking hands, attending gatherings, and hugging of people in direct surrounding area (n = 7,183). CI, confidence interval

### Protective behavior before and after infection or vaccination

**Fig. 3:**
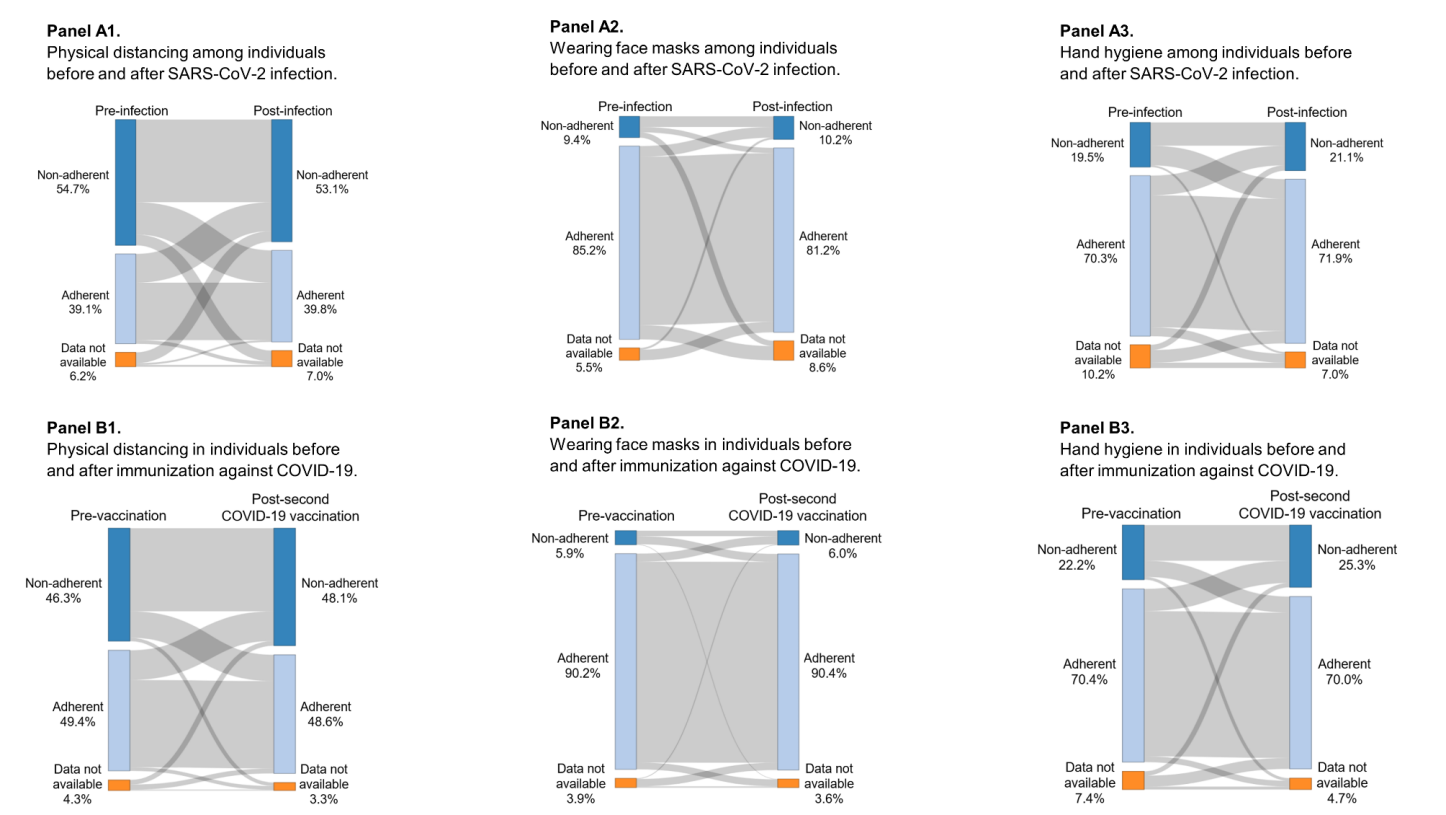
Development of protective behavior before and after infection (Panel A) and vaccination (Panel B). Panel A illustrates the change in protective behavior among individuals before and after SARS-CoV-2 infection. Panel B displays the change in protective behavior before and after vaccination with COVID-19 vaccines

Since protective behavior was assumed to be stable during the pandemic, the development of protective behavior was explored as a function of SARS-CoV-2 infection and COVID-19 vaccination. Adherence to preventive measures in individuals before and after SARS-CoV-2 infection is illustrated in Fig. 3, **Panel A**, and indicates virtually no difference. Immunization with at least two COVID-19 vaccines also did not appear to affect physical distance maintenance, use of face masks, or hand hygiene (Fig. 3, **Panel B**).

## Discussion

The results of this population-based prospective cohort study suggest a preventive effect of physical distancing of 1.5 m and wearing face masks in reducing SARS-CoV-2 infection risk. No beneficial effect was identified for the adherence to hand hygiene measures. The present results indicate similar effects of protective behaviors on viral transmission during the second and third wave of the COVID-19 pandemic, and that these effects applied similarly to individuals of all ages. Interestingly, the adherence to preventive measures was not substantially affected by previous SARS-CoV-2 infection or basic immunization against COVID-19. These findings add to the growing body of evidence on the effect of preventive measures in mitigating the spread of SARS-CoV-2 by showing the impact of protective behaviors with individual-level data of a representative cohort.

### Physical distancing

Consistent with the findings of this study, a large population-based study of 198,022 participants in the US identified physical distancing to reduce SARS-CoV-2 infection risk among individuals living in communities with high adherence to social distancing [8]. A study investigating the effects of various non-pharmaceutical interventions on SARS-CoV-2 transmission in Europe provided further evidence that physical distancing measures are highly effective [21]. A meta-analysis of observational studies additionally found a dose-response relationship between every additional meter of physical distance maintained and reduction in infection risk with SARS-CoV, SARS-CoV-2, and MERS-CoV [10]. In the current study, the effect of physical distancing declined in Cox regression models after adjustment for potential confounders. However, individuals identified as infected only by antibody positivity were excluded from this time-to-event analysis since the date of infection was not known. This reduced the sample size of infected individuals and thus the power to detect a statistically relevant impact.

### Wearing face masks

Regarding the use of face masks, a meta-analysis suggested that face masks largely reduce infection risk of various coronaviruses [10]. However, the certainty of evidence was considered low according to the GRADE guideline, and specifically for SARS-CoV-2, only studies in healthcare settings were considered. In case of community settings, a randomized controlled trial showed that the recommendation to wear face masks did not result in increased protection against SARS-CoV-2 among mask wearers in either the intention-to-treat or per protocol analyses [22]. A systematic review on population-level concluded that using face masks may reduce SARS-CoV-2 infections [23]. However, major limitations were that most of the reviewed studies used mask mandates as exposure, that information on adherence was lacking, and that the effect of other protective measures was mostly not considered. A population-based study further indicated that face mask use reduced SARS-CoV-2 infection risk also among individuals living in communities with poor social distancing [8]. Interestingly, the protective effect against infection was larger in individuals reporting wearing a face mask sometimes (HR = 0.27, 95% CI 0.18–0.41) than among individuals who always wore a mask (HR = 0.38, 95% CI 0.31–0.46). Regarding the effect of masks on transmission of other acute respiratory viruses, a Cochrane review of interventional randomized controlled trials revealed that medical or surgical masks resulted in little or no difference in infection with laboratory-confirmed influenza compared with no masks [24]. This finding was derived from one trial in healthcare workers and five studies in community settings. Similarly, pooled data of four trials in the healthcare sector and one in a household setting additionally indicated no or little difference between N95/FFP2 respirators and medical or surgical masks with respect to infection with laboratory-confirmed influenza. Both findings were considered as moderate-certainty evidence. In a simulation study, the conflicting findings in the literature regarding the efficacy of face masks were explained by the fact that the protective effect depends on airborne virus abundance [25].

Given the current results, adherence to use of face masks was very high in this cohort, limiting the power to detect differences and thus may not reflect the protective effect of face masks. Particularly among women, only one infected individual was identified who never or rarely wore a mask, as a total of only 10 women reported never or rarely wearing a face mask. Hence, after adjustment for sex, the effect of masks was neutralized. Besides, no differentiation was made between the different type of masks (e.g., surgical masks, N95/FFP2 respirators, or self-made masks). Considering the recommendation in the study region Rhineland-Palatinate, it is expected that participants mainly wore surgical masks. Moreover, since the regulation to exclusively wear medical masks became effective on January 25, 2021 in the study region, self-made or cloth masks may have been included in the analyses [26, 27]. The filtration efficacy of these masks is highly dependent on material and number of layers [28], but is still less effective than surgical masks or N95/FFP2 protectors [29]. FFP2 masks have been shown to be the most effective mask with respect to SARS-CoV-2 infection risk [30]. However, regardless of the type of face mask, efficacy strongly depends on the proper fit of the mask. For instance, FFP2 masks whose fit was adjusted with tape around the nose showed the least inward leakage of aerosols. Generally, face masks are medical disposable products that should be changed regularly, especially after moisture intrusion. While FFP2 masks are more resistant and may be reused for private use [31], the reuse of surgical masks is not recommended [32]. The majority of individuals may not sufficiently adjust or appropriately change their face masks, particularly for daily activities such as grocery shopping, which affects the protective performance of masks. The non-distinction of the type of face masks in this study and the reflection of this everyday behavior in the GCS due to its study design may provide additional explanations for the observed result related to face masks and SARS-CoV-2 infection risk.

### Hand hygiene

Hand hygiene has not been identified as a protective factor in reducing SARS-CoV-2 viral transmission. SARS-CoV-2 is primarily transmitted via respiratory droplets and aerosol particles [33, 34], providing a possible explanation for the observed non-protective effect. Consistent with these findings, results from a Swedish population-based study using a similar categorization of handwashing frequency showed no reduced risk of acute respiratory tract infections or influenza-like illness [35]. In contrast, another prospective cohort study suggested a positive effect of moderate frequency of hand washing on acquisition of seasonal coronavirus [11]. However, high frequency of hand hygiene did not result in reduced infection risk and the analysis did not consider other factors of personal risk behavior. Because the evidence on handwashing and influenza-like illness is inconclusive and the evidence on the effect of hand hygiene specifically on SARS-CoV-2 is limited, further research is needed to derive evidence-based recommendations.

Lastly, a simulation study suggested that single preventive measures are not sufficient to substantially reduce highly contagious variants of SARS-CoV-2, such as the Delta or Omicron variants [36]. However, the combination of several measures, such as physical distancing, wearing face masks, frequent testing, and improving the ventilation of rooms, synergistically reduced the reproduction number, considering different scenarios of compliance and vaccination rates. This highlights the importance of implementing and adhering to various public health measures to effectively contain viral transmission.

### Strengths and limitations

**Fig. 4:**
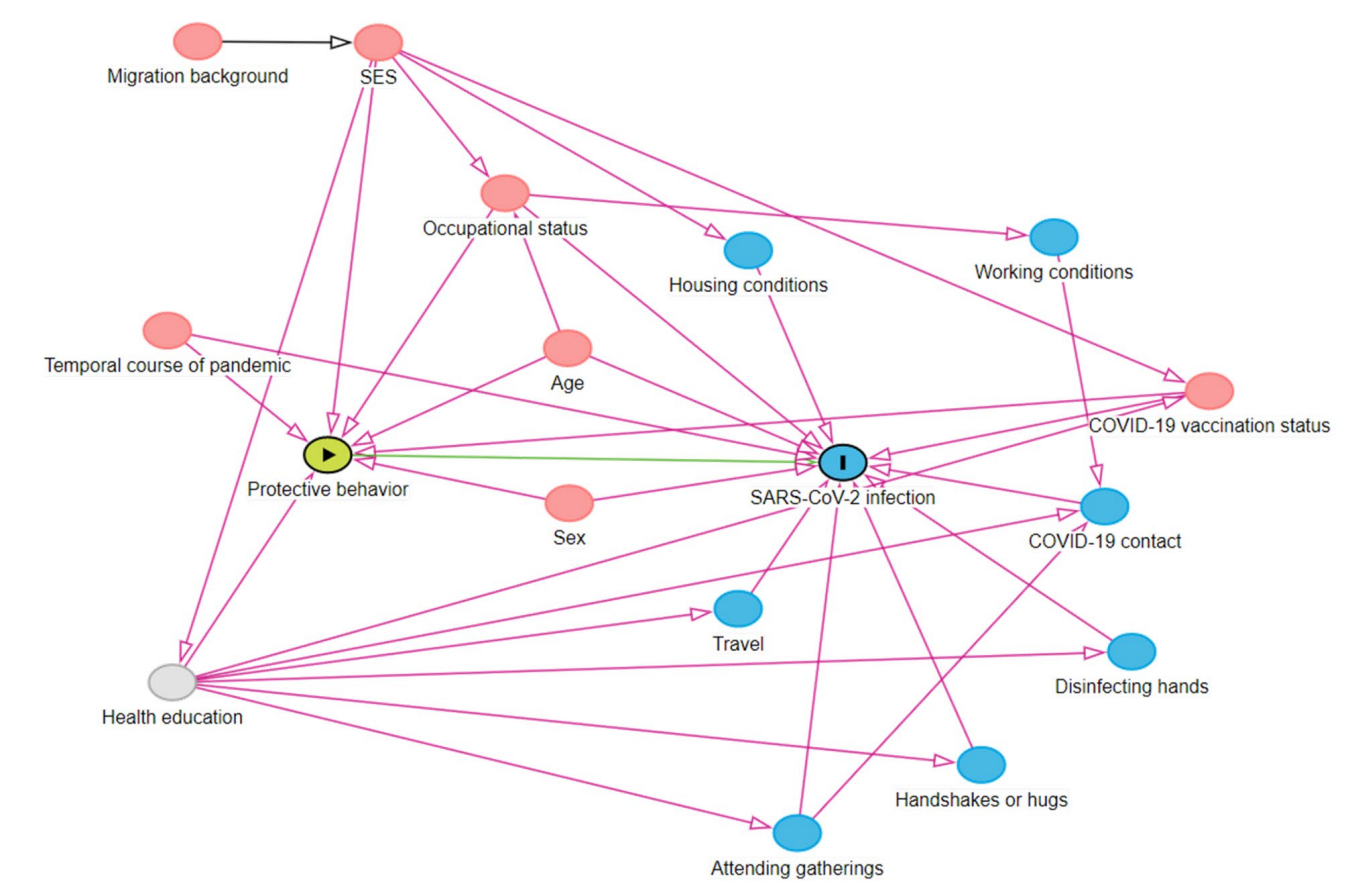
Directed acyclic graph (DAG) of the association between protective behavior and SARS-CoV-2 infection risk. Green: exposure; blue with I: outcome; grey: unobserved variable, blue: ancestor of outcome; red: ancestor of exposure and outcome

The major strength of the present investigation is the large, representative, and comprehensively characterized population sample, which allows adjustment for other public health measures and individual risk behaviors (Fig. 4). Beyond the use of two immunoassays covering different antibody spectra, the GCS included individuals unknowingly infected with SARS-CoV-2, a group of individuals usually not represented or underrepresented in studies investigating SARS-CoV-2. However, some limitations should be noted. Protective behavior at baseline was considered exposure to infection throughout the study period. Therefore, it was assumed that individual protective behavior would not change during the pandemic, and this was demonstrated in the study. In addition, adherence to preventive measures was only self-reported, which could be influenced by recall bias or social desirability [11]. Nevertheless, in the absence of objective measurements for this type of information, self-reported data are currently the best data source, despite the limitations in objectivity.

## Conclusion

The present study suggests physical distancing and wearing face masks to be protective with regard to SARS-CoV-2 infection risk in the general population, supporting their widespread application. The proper fit and use of face masks, however, is extremely important to effectively contain viral transmission. Hand hygiene did not reduce the risk of infection with SARS-CoV-2. Since none of these measures provide complete protection against SARS-CoV-2, other measures such as vaccination and comprehensive testing are still needed to mitigate the spread of SARS-CoV-2. Especially in light of emerging highly contagious variants, such as Omicron, synergistic effects of multiple protective measures are required.

## Electronic supplementary material

Below is the link to the electronic supplementary material.

Supplementary Material 1

## Data Availability

With regard to sharing data of the GCS, study documents, informed consent of the participants, and the approval of the responsible ethics committee do not allow publicly sharing the data according to regulations for data protection (EU General Data Protection Regulation). The data used during the current study are available at the local database from the corresponding author upon request.

## Abbreviations

CI: Confidence interval.
COVID-19: Coronavirus disease 2019.
DAG: Directed Acyclic Graph.
GCS: Gutenberg COVID-19 Study.
GRADE: Grading of Recommendations Assessment, Development and Evaluation.
HR: Hazard ratio.
IQR: Interquartile range.
MERS-CoV: Middle East respiratory syndrome coronavirus.
PR: Prevalence ratio.
RT-qPCR: Quantitative reverse transcription polymerase chain reaction.
SARS-CoV: Severe acute respiratory syndrome coronavirus.
SARS-CoV-2: Severe acute respiratory syndrome coronavirus 2.
SES: Socioeconomic status.
