# Supplementary material for: Protective behavior and SARS-CoV-2 infection risk in the population – Results from the Gutenberg COVID-19 study
Source: BMC Public Health. 2022 Oct 31;22:1993. doi: 10.1186/s12889-022-14310-6 (PMC9623959; doi:10.1186/s12889-022-14310-6)
Supplement: Supplementary file 1 — Supplementary Material 1 [file 12889_2022_14310_MOESM1_ESM.docx]

**SUPPLEMENTARY APPENDIX**

Protective behavior and SARS-CoV-2 infection risk in the population –

Results from the Gutenberg COVID-19 Study

Rieke Baumkötter^1,2^; Simge Yilmaz^1,2^; Daniela Zahn^1^;

Katharina Fenzl^1^; Jürgen H. Prochaska^1,2,3^; Heidi Rossmann^4^; Irene Schmidtmann^5^; Alexander K. Schuster^6^; Manfred E. Beutel^7^; Karl J. Lackner^2,4^; Thomas Münzel^2,8^; Philipp S. Wild^1,2,3,9^

1. Preventive Cardiology and Preventive Medicine, Center for Cardiology, University Medical Center of the Johannes Gutenberg University Mainz, Langenbeckstr. 1, 55131 Mainz, Germany;
2. German Center for Cardiovascular Research (DZHK), Partner Site Rhine Main, University Medical Center of the Johannes Gutenberg University Mainz, Germany;
3. Center for Thrombosis and Hemostasis (CTH), University Medical Center of the Johannes Gutenberg University Mainz, Mainz, Germany;
4. Institute of Clinical Chemistry and Laboratory Medicine, University Medical Center of the Johannes Gutenberg University Mainz, Mainz, Germany;
5. Institute of Medical Biostatistcs, Epidemiology and Informatics, University Medical Center of the Johannes Gutenberg University Mainz, Mainz, Germany;
6. Department of Ophthalmology, University Medical Center of the Johannes Gutenberg University Mainz, Mainz, Germany;
7. Department of Psychosomatic Medicine, University Medical Center of the Johannes Gutenberg University Mainz, Mainz, Germany;
8. Cardiology I, Center for Cardiology, University Medical Center of the Johannes Gutenberg University Mainz, Germany;
9. Institute of Molecular Biology (IMB), Mainz, Germany.

**Correspondence**

Philipp S. Wild, MD, MSc

Professor of Clinical Epidemiology

University Medical Centre Mainz of the Johannes Gutenberg University Mainz, Langenbeckstr. 1, 55131 Mainz, Germany

Phone: +49 6131 17 7163; Fax: +49 6131 17 8460;

Email: philipp.wild@unimedizin-mainz.de

**TABLE OF CONTENTS**

| Identifier | Title | Page |
| --- | --- | --- |
|  |  |  |
| ST | Additional information on PCR and antibody testing | 3 |
|  |  |  |
| S1 | Effect of protective behavior on SARS-CoV-2 infection risk in the population sample. | 4 |
|  |  |  |
| SF1 | Effect of protective behavior on SARS-CoV-2 infections stratified by waves of the SARS-CoV-2 pandemic | 5 |
|  |  |  |
| S2 | Effect of protective behavior on SARS-CoV-2 infection risk according to time periods when different virus variants dominated (February 2020-March 2021 – Wild-type variant; March 2021-June 2021 – alpha variant). | 6 |
|  |  |  |
| SF2 | Effect of protective behavior on SARS-CoV-2 infection risk: interaction with age | 7 |
|  |  |  |
| R | References | 9 |
|  |  |  |

**Supplemental Text**. Additional information on PCR and antibody testing.

*RT-qPCR Testing*

To detect an acute SARS-CoV-2 infection, a swab was taken from the participant’s throat for quantitative reverse transcription polymerase chain reaction (RT-qPCR) analysis. Samples (swabs in 750 µl phosphate-buffered saline) were collected, stored at 4-8°C, and analyzed within 4 days at the latest. For SARS-CoV-2 screening, 200 µl of swab fluid was pooled from each of 5 subjects, RNA was extracted using the QiaAmp UltraSens Virus Kit (Qiagen) and eluted in 30 µl buffer. 10 µl of the eluate were assayed in a 25 µl one-step reverse transcription / qPCR reaction on a cobas z 480 Analyzer (Roche Deutschland Holding GmbH, Germany). Primer and probes for E-gene (plus EAV control) amplification were supplied by TIB MolBiol (Light Mix SarbecoV, TIB Molbiol, Germany), reagents for reverse transcription and qPCR by Qiagen (One-Step QuantiTect Probe RT-PCR Kit, Qiagen, Germany). Details on assay implementation, quality and quality control are described in Hauser et al. (1, methods: ‘laboratory 2’). In case of a positive pool, all samples were re-tested individually by E-gene and RdRP-gene (Light Mix SarbecoV, TIB Molbiol, Germany) qPCR as described in Hauser et al. [1; methods: ‘laboratory 2’].

*SARS-CoV-2 Antibody Testing*

EDTA samples were analyzed regarding circulating antibodies targeted at SARS-CoV-2 nucleocapsid with a qualitative microparticle chemiluminescent immunoassay (Architect SARS-CoV-2 IgG, Abbott, Germany) with a threshold of 1.4 relative light units and second, a qualitative microparticle electro-chemiluminescence immunoassay (Elecsys Anti-SARS-CoV-2 Pan-Ig, Roche, Germany) with a cutoff index of 0.8. Samples were collected and stored at room temperature and were analyzed with the Abbott immunoassay within two days at the latest. Subsequently, samples were stored at −80°C in a state-of-the-art biobanking facility at the University Medical Center Mainz. After defrosting the samples, they were re-analyzed with the immunoassay of Roche.

**Supplemental Table 1.** Effect of protective behavior on SARS-CoV-2 infection risk in the population.

|  | Univariate analysis* | | Adjusted for time of enrollment, sociodemographics, pandemic-related behavior^†^ | |
| --- | --- | --- | --- | --- |
|  | Hazard ratio [95% CI] | *P* | Hazard ratio [95% CI] | *P* |
|  |  |  |  |  |
| Physical distancing | 0.56 [0.40; 0.77] | 0.00052 | 0.66 [0.43; 1.02] | 0.058 |
| Use of face masks | 0.91 [0.53; 1.58] | 0.74 | 1.22 [0.63; 2.37] | 0.56 |
| Hand hygiene | 1.09 [0.74; 1.60] | 0.67 | 1.14 [0.72; 1.82] | 0.58 |

Cox regression models. Dependent variable: SARS-CoV-2 infections since January 2020 (individuals without date of infection were excluded); independent variables: physical distancing, wearing face masks, and hand hygiene at baseline.

* physical distancing (n=9,344, 155 events), use of face masks (n=9,362, 156 events), hand hygiene (n=9,016, 145 events).

^†^ Adjustment for time of enrolment into study, age, sex, socioeconomic status, occupational

status, COVID-19 vaccination status, COVID-19 contact in the past two weeks, participation in gatherings in the past two weeks, travel (any), disinfecting hands (>2 per day), and avoidance of shaking hands, attending gatherings, and hugging of people in direct surrounding area (n=6,945, 111 events). CI, confidence interval.

**Supplemental Figure 1.** Effect of protective behavior on SARS-CoV-2 infections stratified by waves of the SARS-CoV-2 pandemic.


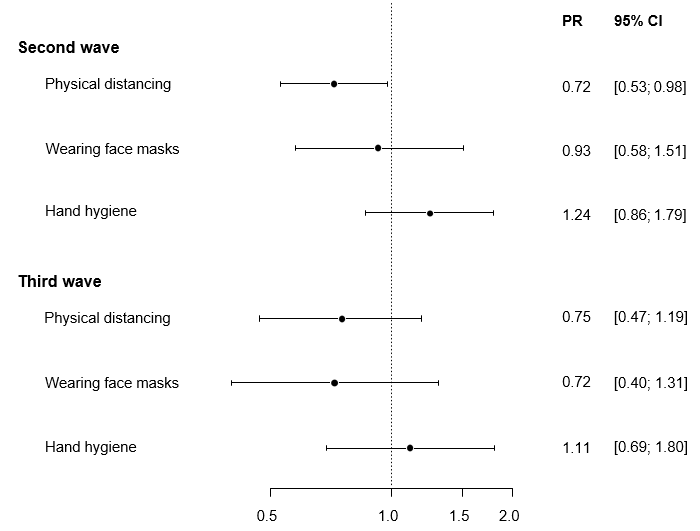


Prevalence ratio

Presented are prevalence ratios of multivariable robust Poisson regression models.

Second wave of the SARS-CoV-2 pandemic in Germany: September 28, 2020 to February 28, 2021 (n=7,016). Third wave of the SARS-CoV-2 pandemic in Germany: March 1, 2021 to June 13, 2021 (n=6,928).

Dependent variable: SARS-CoV-2 infections during baseline and follow-up; independent variables: physical distancing, wearing face masks, and hand hygiene at baseline.

Adjustment for time of enrollment into study, age, sex, socioeconomic status, occupational status, COVID-19 vaccination status, COVID-19 contact in the past two weeks, participation in gatherings in the past two weeks, travel (any), disinfecting hands (>2 per day) and avoidance of shaking hands, attending gatherings, and hugging of people in direct surrounding area. PR, prevalence ratio; CI, confidence interval.

**Supplemental Table 2.** Effect of protective behavior on SARS-CoV-2 infection risk

according to time periods when different virus variants dominated

(February 2020-March 2021– Wild-type variant; March 2021-June

2021 – alpha variant).

|  | February 2020-March 2021 | | March 2021-June 2021 | |
| --- | --- | --- | --- | --- |
|  | Prevalence ratio [95% CI] | *P* | Prevalence ratio [95% CI] | *P* |
| Physical distancing | 0.78 [0.59; 1.04] | 0.089 | 0.81 [0.60; 1.10] | 0.17 |
| Use of face masks | 0.85 [0.54; 1.32] | 0.46 | 1.00 [0.62; 1.59] | 0.99 |
| Hand hygiene | 1.08 [0.78; 1.50] | 0.64 | 1.17 [0.83; 1.64] | 0.38 |

February 2020-March 2021: time period in which the wild type was dominant in Germany (N=7,804, n=251 events). March 2021-June 2021: time period in which alpha was the dominant strain in Germany (N=7,797, n=250 events) [2]. Robust Poisson regression with adjustment for age, sex, SES, occupational status. CI, confidence interval.

**Supplemental Figure 2.** Effect of protective behavior on SARS-CoV-2 infection risk: interaction with age.

**Panel A.** Physical distancing


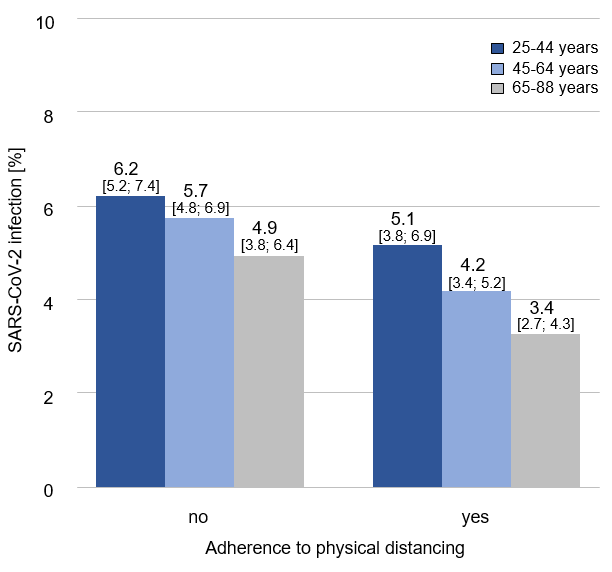


*P* for interaction = 0.81

**Panel B.** Wearing face masks


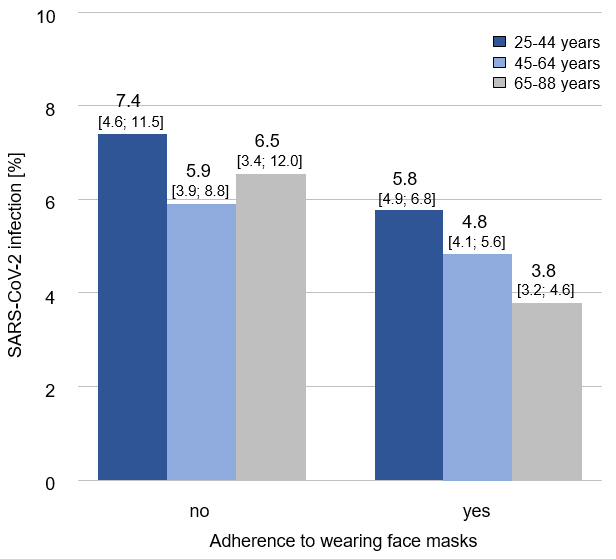


*P* for interaction = 0.74

**Panel C.** Hand hygiene


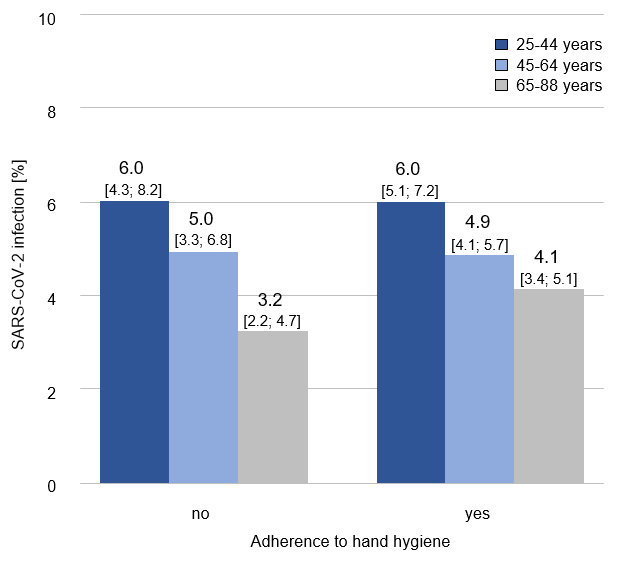


*P* for interaction = 0.93

**References**

1. Hauser F, Sprinzl MF, Dreis KJ, Renzaho A, Youhanen S, Kremer WM, et al. Evaluation of a laboratory-based high-throughput SARS-CoV-2 antigen assay for non-COVID-19 patient screening at hospital admission. Med Microbiol Immunol. 2021;210(2-3):165-71.

2. Robert Koch Institut (RKI). Bericht zu Virusvarianten von SARS-CoV-2 in Deutschland. 2021.
